# Melatonin—A New Prospect in Prostate and Breast Cancer Management

**DOI:** 10.7759/cureus.18124

**Published:** 2021-09-20

**Authors:** Comfort Anim-Koranteng, Hira E Shah, Nitin Bhawnani, Aarthi Ethirajulu, Almothana Alkasabera, Chike B Onyali, Jihan A Mostafa

**Affiliations:** 1 Medicine, California Institute of Behavioral Neurosciences & Psychology, Fairfield, USA; 2 Internal Medicine, California Institute of Behavioral Neurosciences & Psychology, Fairfield, USA; 3 General Medicine, California Institute of Behavioral Neurosciences & Psychology, Fairfield, USA; 4 Internal Medicine, EHA Clinics, Abuja, NGA; 5 Psychiatry, California Institute of Behavioral Neurosciences & Psychology, Fairfield, USA

**Keywords:** antiproliferation, vegf angiogenesis, chemotherapy, melatonin, breast cancer, prostate cancer, radiotherapy (rt)

## Abstract

Cancer is a known cause of mortality globally. The management of cancer has been influenced periodically by diverse scientific research for early detection to promote remission and improve quality of life. One of these advancements is the prospect of melatonin (n-acetyl-5-methoxytryptamine) in managing prostate and breast cancers. Melatonin exerts its oncostatic effect by inhibiting angiogenesis, preventing cancer spread and growth, and improving the sensitivity of cancer cells to radiation and chemotherapy in both prostate and breast cancer. This review aims to highlight some of the current studies on melatonin's effect on prostate and breast cancers. We reviewed articles and two randomized controlled trials (RCT) that highlighted the mechanism of melatonin in combating tumorigenesis of these cancers. Articles and RCT studies were obtained by searching PubMed using regular and Medical Subject Heading (MeSH) keyword search strategy. The majority of the articles reviewed supported the use of melatonin in cancer management since inhibition of angiogenesis, cancer proliferation, invasion of normal cells by tumor cells, and improvement in chemotherapeutic and radiation therapy were achieved with its use. In addition, melatonin was also protective against prostate and breast cancers in the general population. Despite the benefits of melatonin in cancer management, most of the studies done were in vivo and in vitro studies, and more studies in human subjects are encouraged to confirm the positive therapeutic use of melatonin.

## Introduction and background

Universally, cancer is the second leading cause of death. Its incidence is projected to increase to 19.3 million annually by 2025 [1]. According to the Center for Disease Control and Prevention (CDC), prostate cancer (PC) and breast cancer (BC) are the most common malignancies among men and women, respectively, in the United States of America (USA), aside from non-melanoma skin cancer [2]. Approximately 12.5% of men will be diagnosed with prostate cancer [2], while 12.9% of women will be diagnosed with breast cancer during their lifetime [3]. Though breast cancer-related mortality has declined, it is still the second leading cause of death among women, as prostate cancer is among men. However, most men diagnosed with prostate cancer do not die from cancer but have a significant reduction in health-related quality of life [4].

The management of prostate and breast cancer has evolved over the decades, and like most malignancies, management is based on the stage. The low-risk localized prostate lesion is either managed surgically (prostatectomy) or with radiation therapy. In contrast, the high-risk localized lesion is managed with radiation therapy and adjunct androgen deprivation therapy (ADT) either by luteinizing hormone-releasing hormone (LHRH) agonist or antagonist or surgical castration [5]. Advanced prostate cancer is managed with ADT, with or without chemotherapy. Unfortunately, most patients with advanced prostate cancer progress to ADT resistance over a period [6]. With ADT being the standard of care in prostate cancer [7], alternatives or adjuncts to ADT must be incorporated in management to reduce this resistance progression and enhance efficacy. The current therapies for breast cancer include surgery, chemotherapy, hormonal, biological, and radiation therapy [8].

The quest for anticancer agents from natural products to supplement current therapy has led to the discovery of anticancer properties of melatonin, which have been studied over decades [9].

**Figure 1:**
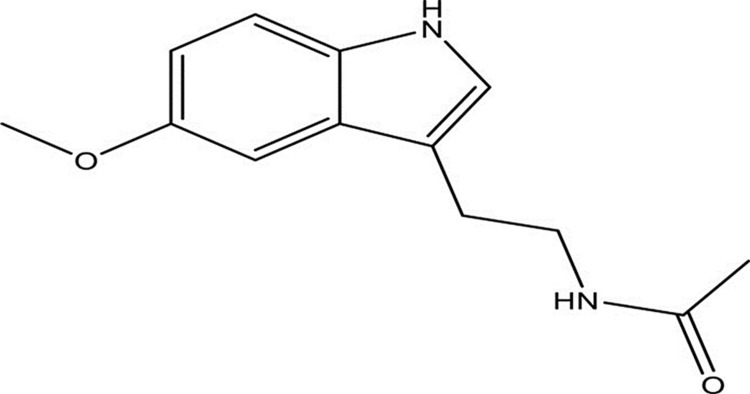
Structure of melatonin

Melatonin (n-acetyl-5-methoxytryptamine, as in Figure 1) is an indolic compound synthesized in diverse cells and organs in the body such as the gastrointestinal tract (GIT), bone marrow, membranous cochlea, several leucocytes, and other regions of the leucocytes, aside from the pineal gland [4]. The release of this neurohormone is regulated by the circadian cycle, seasons, gender, and other physiological conditions [7,8]. The liver metabolizes melatonin via the cytochrome P450 pathway with 6-hydroxy-melatonin (6-OHM) as its metabolite [9].

Melatonin has ubiquitous functions outside its role in the circadian rhythm [10]. These include antioxidant effects [10], improvement in depression and anxiety [11], and anticancer effects [9]. Melatonin exerts its anticancer activities via receptor-dependent and receptor-independent mechanisms [9]. The receptor-dependent mechanism inhibits adenyl cyclase and cyclic adenosine monophosphate (cAMP), which declines guanine protein receptor-mediated linoleic acid uptake. The receptor-independent mechanism leads to an antiproliferative effect on cells, antioxidant activity, regulation of cell death, tumor metabolism and cancer immunity, inhibition of angiogenesis and migration, and prevention of circadian disruption [12-14].

A dose of melatonin within the nanomolar (nM) range (≤10^-9 ^M) usually leads to cytostatic effects, while apoptosis occurs at higher concentrations [15]. At a dose of 10 nM, it demonstrates antiproliferative effects, while at 50 nM, it influences cell growth, and at 1 millimolar (mM), it influences cell viability. Unlike the traditional chemotherapeutic agents, melatonin induces death only in cancerous cells sparing the normal cells [15,16]. In addition, melatonin administration at various doses from 1 mg to 10 mg/kg, either as short, intermediate, or long-term administration, is shown to offer very minimal side effects compared to medications serving a similar purpose as melatonin. Mild adverse effects reported include headache, nausea, sleepiness, and dizziness [17].

This article highlights the current studies on melatonin's oncostatic effect on prostate and breast cancer [9] since these are the most common cancers aside from skin cancer in both males and females.

## Review

Melatonin’s oncostatic effect on prostate cancer

The increasing complexities of long-term cancer treatments have become challenging for both patients and physicians. As such, cancer prevention has been identified as a vital control strategy with lifestyle modification to eliminate modifiable risk factors and screening being critical to early detection [18]. Clinical trials have demonstrated the efficacy of some medications in the prevention of prostate cancer. The Prostate Cancer Prevention Trial (PACT) demonstrated that finasteride is associated with reducing the risk of prostate cancer. In this study, 10.5% of participants in the finasteride group compared to 14.9% of the placebo group developed prostate cancer (relative risk [RR] in the finasteride group: 0.70; 95% confidence interval [CI]: 0.65 to 0.76; *P* < 0.001). However, 3.5% in the finasteride group and 3.0% in the placebo group who developed prostate cancer had high-grade cancer with a Gleason score of 7 to 10 (RR: 1.17; 95% CI: 1.00 to 1.37; *P *= 0.05). Finasteride was also associated with side effects like erectile dysfunction, decreased libido, and gynecomastia [19]. The Reduction by Dutasteride of Prostate Cancer Events (REDUCE) trial also demonstrated protection against prostate cancer in men who took dutasteride compared to the placebo but with side effects [20]. These side effects may limit the impact of these medications on prostate cancer prevention, and medication with similar efficacy without these limiting side effects will be a game-changer. Melatonin is proven to lower the risk of prostate cancer [21] with minimal to no side effects [17]. Therefore, it can be useful in prostate cancer prevention. An epidemiological study done in Iceland between 2002 and 2009 demonstrated that men with lower first-morning 6-sulfatoxymelatonin (aMT6) below the median had a four-fold statistically significant higher risk for advanced prostate cancer than men with higher levels (hazard ratio [HR]: 4.04; 95 % CI: 1.26-12.98) [21]. Another case-control study done from 2011 to 2014 showed a similar result where men with higher urinary melatonin-sulfate were less likely to develop prostate cancer (adjusted odds ratio [aOR]: 0.59, 95% CI: 0.35-0.99) or advanced prostate cancer (aOR: 0.49; 95% CI: 0.26-0.89) [22]. It is important to note that while the incidence of prostate cancer was reduced in both the PACT and REDUCE trials, there was an increased incidence of high-grade prostate cancer with finasteride compared to placebo. Dutasteride in the REDUCE study did not statistically influence the incidence of advanced prostate cancer compared to placebo [20]. Melatonin, however, was found to statistically reduce the incidence of advanced prostate cancer [22,23]. This could demonstrate a competitive edge of melatonin over these conventional therapies but more studies will be essential to compare the protective effect of finasteride, dutasteride, and melatonin on prostate cancer. Hormonal therapy is the cornerstone in PC management since androgens (testosterone and dihydrotestosterone [DHT]) stimulate PC cells to grow. PC cell lines undergo modifications when exposed to hormonal therapy with time, leading to refractory cancer in most people [24]. The progression to castration-resistant prostate cancer (CRPC) occurs within two to three years of initiation of ADT [7], which is very concerning for a disease that is the second leading cause of mortality among men in the USA. Docetaxel has been the mainstay of treatment for patients with CRPC since its approval in 2004, but CRPC patients develop resistance to docetaxel or may not tolerate the side effects, limiting its use [25]. The rising incidence of CRPC led to the emergence of newer medications like abiraterone and sipuleucel-T for the management of CRPC but has not been cost-effective [26]. Therefore, it is urgent to find new cost-effective therapeutic alternatives with fewer side effects. A mechanistic study revealed that melatonin reduces PC progression to CRPC by decreasing lipid accumulation and activity in PC cells via upregulating the lipid metabolism-related gene carboxylesterase (CES) 1 in PC cells [27].

The use of melatonin with conventional PC therapy has shown therapeutic benefits. A recent retrospective study in Russia that spanned 20 years assessed the effect of melatonin on the survival rate of prostate cancer patients with varied prognoses. Out of 955 participants, 113 had a favorable prognosis, 187 had an intermediate, and 655 had a poor prognosis. All participants received combined hormonal and radiation therapy with or without 3 mg of melatonin daily. The study revealed a statistically significant improvement in the five-year survival rate (153.5 months vs. 64 months; *P *< 0.0001) of those with poor prognosis treated with melatonin compared to those not treated with melatonin. However, there was no statistically significant improvement among the patients with favorable and intermediate prognoses [28]. This reflects the beneficial effects of melatonin in advanced prostate cancer patients with poor prognosis, and more research is encouraged to investigate when it is feasible to start melatonin treatment in those with intermediate prognosis who might progress to poor prognosis.

Melatonin has also been shown to reduce proliferation and angiogenesis and inhibit migration of cancer cells. A study was done by Calastretti et al. using melatonin analog UCM 1037 on androgen-sensitive prostate cell lines (LNCaP and 22Rv1) and androgen insensitive prostate cell lines (DU145 and PC3) demonstrated a significant reduction of proliferation in the androgen-sensitive prostate cell lines. These cell lines were seeded in a 96-well plate and treated with UCM 1037 at 10^-6^-10^-4^ M after 24 hours or received melatonin at 10^-5^-10^-3^ M in 0.1% dimethyl sulfoxide (DMSO) for 24, 48, and 72 hours while the control received 0.1% DMSO at the exact timelines. The result is demonstrated in Table 1 below.

**Table 1:** Reduction of cell line numbers when treated with UCM 1037

Cell line + UCM 1037 10^-4 ^M	Cell numbers after 48 hours	Cell numbers after 72 hours
LNCaP	38%	34%
22Rv1	31%	14%
DU145	73%	67%
PC3	80%	68%

The study revealed that most melatonin receptor-1 (MT1) were found in LNCaP and less in DU145, 22Rv1, and PC3, while melatonin receptor-2 (MT2) was found only in the androgen insensitive cell lines [29]. This study showed a significant reduction in the growth of androgen-sensitive prostate cancer cells when treated with melatonin derivatives. This demonstrates melatonin’s potential therapeutic effect on androgen-sensitive prostate cancers.

Angiogenesis is critical for the growth of tumor cells, and melatonin is known to exert an anti-angiogenetic effect on cancer cells. In an in-vivo study by Paroni et al., LNCaP cell lines were xenografted into seven-week-old Foxn1/nu male mice, with the test mice treated with 18 intraperitoneal injections of 1 mg/kg melatonin for 41 days while the control mice received saline. Seventy-two hours after the last melatonin administration, there was evidence of lower micro-vessel density in the test mice compared to the control with a four-fold reduction in cancer size. This anti-angiogenic effect was achieved by decreased expression of nuclear protein Ki67, increased hypoxia-inducible factor (HIF)-1 alpha expression, and protein kinase B (Akt) phosphorylation [30]. Melatonin also expresses its anti-angiogenic effect by upregulating microribonucleic acid (miRNA) such as miRNA 3195 and miRNA 374B in a prostate cell line (PC-3) under hypoxic conditions as they influenced mRNA levels of angiogenesis-related proteins HIF-1 alpha, HIF-2 alpha, and vascular endothelial growth factor (VEGF) [31].

Melatonin inhibits migration and invasion of prostate cancer cells to surrounding cells by interacting with MT1 receptor, inhibiting other signaling pathways like phospholipase C (PLC), protein kinase 38 (p38), and c-Jun dependent pathways in DU145 and PC3 prostate cancer cell lines. In addition, melatonin reduces the metastatic ability of PC by reducing the expression of metalloproteinase 13 (MMP-13) in both in vivo and in vitro models [23].

Melatonin's oncostatic effect on breast cancer

The level of melatonin in blood has been shown to correlate to a reduced risk for breast cancer. In one dose-response analysis of published observational studies, levels of serum melatonin (>39.5 pg/mL), as well as its primary metabolite aMT6 of 15 ng/mg creatinine, is associated with a reduced risk of breast cancer by 14 % (RR: 0.86, 95% CI: 0.78-0.95) compared to subjects with lower levels [32]. Exposure to artificial light at night, especially in night-shift workers, is known to increase the risk for breast cancer (BC) due to disruption of the circadian rhythm with decreased melatonin production, though other studies conclude otherwise [32,33]. In a recent meta-analysis study, night work was found to be associated with an increased risk of BC (RR: 1.2, 95% CI: 1.08-1.33) [33], highlighting the protective effect of melatonin against breast cancer [34].

Clinical trials in women with high risk for BC have demonstrated that treatment with tamoxifen, raloxifene, anastrozole, and exemestane for five years reduces the incidence of estrogen receptor (ER) positive BC but not of ER-negative tumors [35]. However, the side effects of hot flushes, thromboembolic phenomenon, and increased risk of endometrial cancer have limited use of these medications [36]. Melatonin has been demonstrated in experimental studies to potentiate the efficacy of these medications while reducing the side effects [37].

Recent studies have demonstrated a remarkable synergistic effect of melatonin with chemotherapy and radiotherapy [9]. A seven-day pretreatment of MCF-7 breast cell lines with melatonin (1 mm, 10 mcm, and 1 nm) before radiation led to an alteration in the phases of the cell cycle (decreased G2-M phase arrest, more cells remaining in G0-G1 phase, and a lower percentage of cells in S phase) and downregulation of RAD51 recombinase (RAD51) and DNA protein kinase (PKcs) mRNA (proteins involved in double-strand DNA break repair) compared to treatment with radiation alone. This demonstrates that melatonin increases the sensitivity of cells to radiation therapy and improves efficacy [9]. In addition, melatonin improved the pharmacodynamics of doxorubicin by activating transient receptor potential vanilloid 1 (TRPV1), apoptosis, and inducing MCF-7 human breast cancer cell death [38]. Studies on MCF-7 human breast cell line grown in rats revealed that a disruption of the circadian rhythm by dim light exposure at night (dLEN) resulted in less melatonin production and induced intrinsic resistance to Erα+ MCF-7 breast cell lines treated with doxorubicin and tamoxifen. Reintroduction of nocturnal melatonin improved the sensitivity of the breast cell lines to doxorubicin and tamoxifen and tumor suppression in both studies [39,40]. Thus, it can be stipulated that light exposure at night can be a risk factor to specific chemotherapeutic resistance, and melatonin supplementation while on cancer treatments could make these therapies more efficacious by reducing intrinsic resistance.

Melatonin is also known to regulate miRNA and subsequently suppress tumor metastasis [41,42]. Microribonucleic acids (miRNAs) are small molecules of RNA involved in the post-transcriptional level (non-coding) of gene expression and associated with the metastatic process, tumor suppression, and oncogenesis as it plays crucial roles in these processes when deregulated [43-46]. In a study involving canine and human cell lines, melatonin was shown to prevent the spread of breast cancer by reducing cell viability and invasiveness and enhancing the expression of epithelial-mesenchymal transition (EMT)-related proteins [47]. This anti-invasive effect was also demonstrated by downregulation of the p38 pathway and suppression of matrix metalloproteinase 2 (MMP-2) and matrix metalloproteinase 9 (MMP-9) expressions and activity [48].

Melatonin also demonstrates its antiproliferative and apoptotic effects in the breast cancer cell (MDA-MB-361) by simultaneous modulation of cyclooxygenase-2 (COX-2)/prostaglandin E2 (PGE2), p300/nuclear factor kappa B (NF-KB), phosphoinositide 3-kinase (PI3K)/protein kinase B (Akt) signaling pathway, and activation of the apoptotic protease activating factor 1 (Apaf-1)/caspase-dependent apoptotic pathway at pharmacological concentrations of 10⁻³ m [49]. Other studies have indicated a similar antiproliferative effect of melatonin at doses of 1 mM, 10-40 mg/kg on breast cancer via varied mechanisms [37,50-53].

Limitations

This article has some limitations in that most of the studies were done in vitro and in vivo, with limited data on clinical trials in humans. Melatonin's oncostatic effect has been studied over decades so most of the studies available were decades old, and only a few recent studies were available.

## Conclusions

Melatonin is a neurohormone produced from other sites of the body aside from the pineal gland. Research studies have revealed the diverse use of melatonin in addition to its regulation of the circadian rhythm. This article has demonstrated its potential therapeutic use in reducing prostate and breast cancer risk and as an adjunct to both chemotherapy and radiation therapy with improved efficacy and probable minimal side effects. However, the majority of the studies were in vitro and in vivo, and hence more clinical trials are needed to assess these therapeutic benefits and side effects in the human population.
